# A General Method for the Synthesis of Hybrid Nanostructures Using MoSe_2_ Nanosheet-Assembled Nanospheres as Templates

**DOI:** 10.34133/2019/6439734

**Published:** 2019-11-14

**Authors:** Shikui Han, Kai Zhou, Yifu Yu, Chaoliang Tan, Junze Chen, Ying Huang, Qinglang Ma, Ye Chen, Hongfei Cheng, Weijia Zhou, Hua Zhang

**Affiliations:** ^1^Key Laboratory of Advanced Catalytic Materials and Reaction Engineering, School of Chemistry and Chemical Engineering, Hefei University of Technology, Hefei 230009, China; ^2^Center for Programmable Materials, School of Materials Science and Engineering, Nanyang Technological University, 50 Nanyang Avenue, Singapore 639798; ^3^Center for Advanced Analytical Science, School of Chemistry and Chemical Engineering, Guangzhou University, Guangzhou 510006, China; ^4^New Energy Research Institute, School of Environment and Energy, South China University of Technology, Guangzhou Higher Education Mega Center, Guangzhou 510006, China; ^5^Department of Chemistry, City University of Hong Kong, Tat Chee Avenue, Kowloon, Hong Kong

## Abstract

The layered transition metal dichalcogenides (TMDs) and transition metal phosphides are low-cost, earth-abundant, and robust electrocatalysts for hydrogen evolution reaction (HER). Integrating them into hybrid nanostructures is potentially promising to further boost the catalytic activity toward HER based on their synergistic effects. Herein, we report a general method for the synthesis of a series of MoSe_2_-based hybrid nanostructures, including MoSe_2_-Ni_2_P, MoSe_2_-Co_2_P, MoSe_2_-Ni, MoSe_2_-Co, and MoSe_2_-NiS, by postgrowth of Ni_2_P, Co_2_P, Ni, Co, and NiS nanostructures on the presynthesized MoSe_2_ nanosheet-assembled nanospheres, respectively, via a colloidal synthesis method. As a proof-of-concept application, the as-synthesized hybrid nanostructures are used as electrocatalysts for HER, exhibiting high activity and stability in acidic media. Among them, the MoSe_2_-Co_2_P composite shows the highest HER activity with an overpotential of 167 mV at 10 mA cm^−2^.

## 1. Introduction

With increasing concerns on the global environmental contamination and energy shortage caused by the excessive consumption of fossil fuels, hydrogen as a promising chemical fuel has been considered as a clean and sustainable alternative [1–4]. However, the massive and sustainable hydrogen production from the electrocatalytic water splitting requires highly efficient and robust catalysts [5–9]. Although platinum (Pt) and other precious metals have shown superior catalytic performance in the hydrogen evolution reaction (HER) at low overpotentials in acidic media, their scarcity and high cost limit their practical applications [10–12]. Therefore, it is still a great challenge to find cost-effective and earth-abundant electrocatalysts with high HER activity, low overpotential, and excellent stability to replace the rare and expensive noble metal electrocatalysts.

**Scheme 1:**
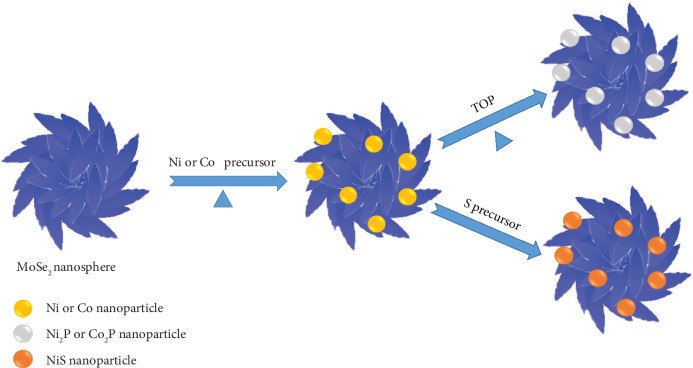
Schematic illustration of synthesis of the MoSe_2_ nanosphere-based hybrid nanostructures.

Among various non-noble-metal HER catalysts, transition metal semiconductor nanomaterials, e.g., dichalcogenides and phosphides, have been extensively studied [13–21], because of their low cost, high abundancy, and high HER catalytic activity [22–28]. Recently, theoretical calculations and experimental studies on MoS_2_ nanosheets suggested that the exposed edge is one of the catalytically active sites for hydrogen evolution [29, 30], which inspired researchers to prepare edge-rich MoS_2_ nanostructures to enhance the HER performance [31–34]. Furthermore, based on the density functional theory, Tsai et al. found that the HER performance of MoSe_2_ can be comparable to or even better than that of MoS_2_ [35]. However, there are only a few reports on designing the catalytically active MoSe_2_ nanostructures for HER [36–41]. Besides transition metal dichalcogenides (TMDs), the emerging transition metal phosphides have also drawn extensive attention as effective HER catalysts due to their good durability, corrosion resistance, and high current density at low overpotential [42–50]. Among them, the Co- and Ni-based phosphide nanostructures have exhibited their great potential as the electrocatalysts for HER [26, 51–54]. Therefore, it is very important to design and synthesize MoSe_2_-based hybrid HER electrocatalysts by combining the advantages of both MoSe_2_ and transition metal phosphides. Herein, we report a general colloidal method for the synthesis of a series of hybrid nanostructures using the MoSe_2_ nanosheet-assembled nanospheres as templates, including MoSe_2_-Ni_2_P, MoSe_2_-Co_2_P, MoSe_2_-Ni, MoSe_2_-Co, and MoSe_2_-NiS (Scheme 1). First, MoSe_2_ nanospheres were prepared by a hot-injection method. Then, the obtained MoSe_2_ nanospheres mixed with transition metal cations, oleylamine, and trioctylphosphine in a three-necked flask. Because the surface potential of the freshly prepared MoSe_2_ nanospheres is negative, the transition metal cations in the solution can be easily adsorbed on the surface of MoSe_2_ nanosheets via the electrostatic interaction. As shown in Scheme 1, when the reaction temperature was increased to about 220°C, Ni or Co nanoparticles were formed. If the reaction temperature was further increased to 320°C, the trioctylphosphine would react with transition metals to form the transition metal phosphides on MoSe_2_ nanospheres. However, if the reaction temperature was 220°C, after addition of the S precursor, the transition metal sulfide, such as NiS, nanoparticles were formed on MoSe_2_ nanospheres. As a proof-of-concept application, the as-prepared hybrid nanostructures exhibit high electrocatalytic HER activity and stability in acidic media.

## 2. Results

**Figure 1:**
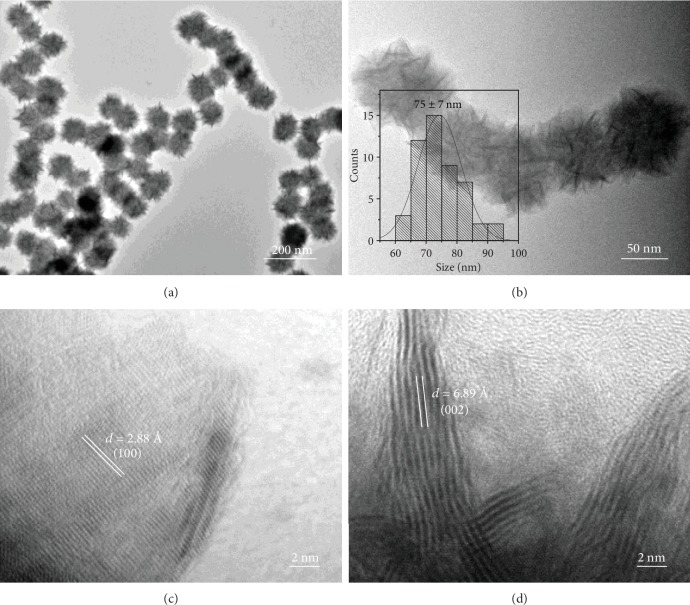
TEM and HRTEM measurements of the as-prepared MoSe_2_ nanospheres. (a, b) TEM images and (c, d) HRTEM images. Inset in (b): statistical analysis of the size of 50 MoSe_2_ nanospheres measured from TEM images.

Briefly, the colloidal MoSe_2_ nanosheet-assembled nanospheres were prepared by injection of the selenium-octadecene precursor into a mixture of octadecene and stearic acid containing MoCl_5_ at 300°C, which was kept for 30 min (see Materials and Methods for details). The morphologies of the obtained colloidal MoSe_2_ nanospheres were characterized by a transmission electron microscope (TEM). As shown in Figures 1(a) and 1(b), the prepared MoSe_2_ nanospheres with size of 75 ± 7 nm (inset in Figure 1(b)) were formed by the assembly of MoSe_2_ nanosheets. High-resolution TEM (HRTEM) image of a typical edge of MoSe_2_ nanosheet confirms its single-crystalline nature (Figure 1(c)). The lattice distances of 2.88 Å and 6.89 Å can be assigned to the (100) and (002) planes of 2H phase MoSe_2_ [37], respectively (Figures 1(c) and 1(d)). The powder X-ray diffraction (XRD) pattern ([Supplementary-material supplementary-material-1]) further confirms that the nanosphere consists of crystalline 2H phase MoSe_2_ (JCPDS No. 15-0029, hexagonal, *a* = 3.288 *Å*, *c* = 12.89 *Å*). As shown in [Supplementary-material supplementary-material-1], the peaks located at 31.69°, 37.38°, and 56.10° correspond to the (100), (103), and (110) planes of the hexagonal MoSe_2_, respectively.

**Figure 2:**
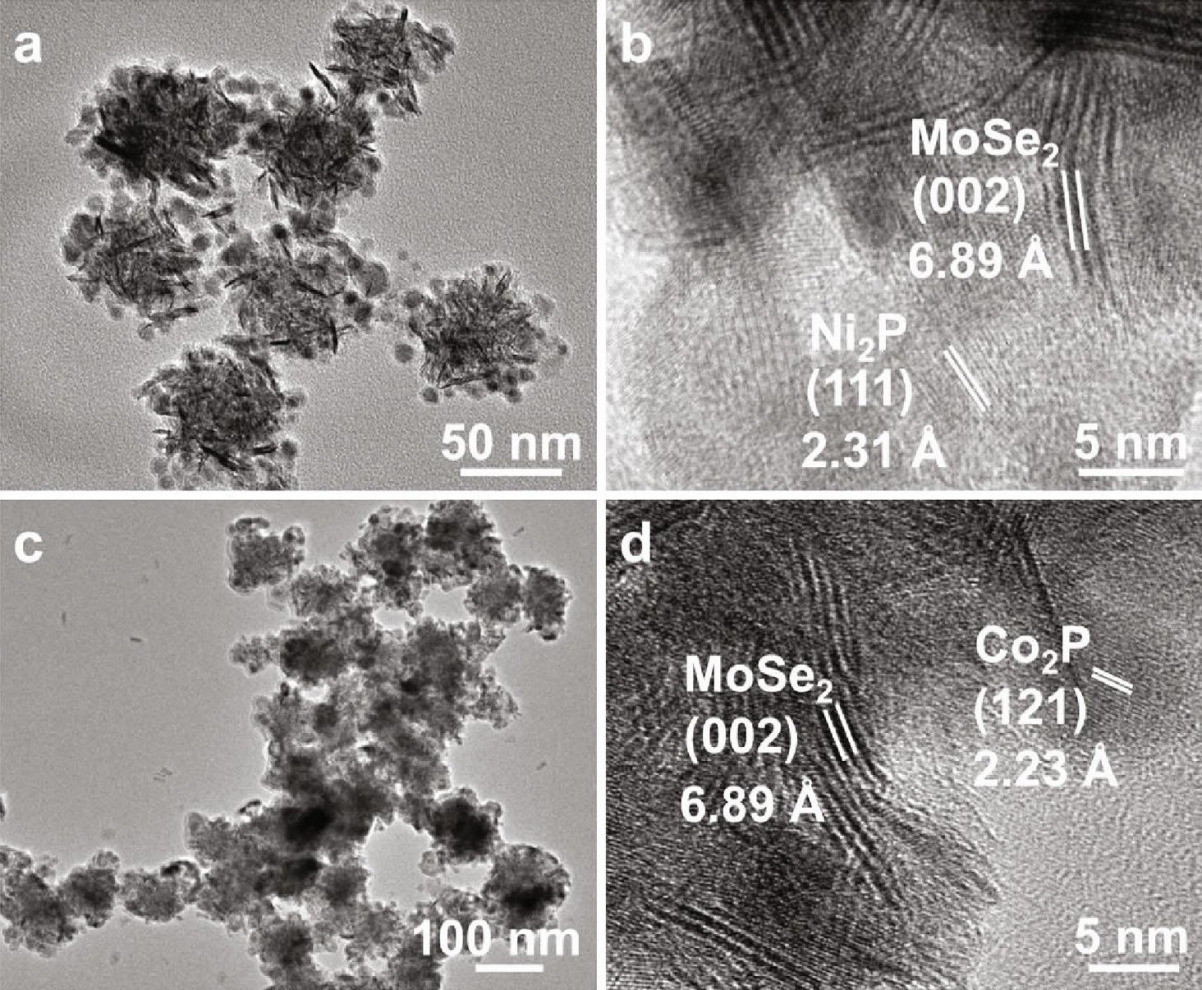
TEM and HRTEM measurements of the MoSe_2_-Ni_2_P and MoSe_2_-Co_2_P hybrid nanostructures. (a) TEM and (b) HRTEM images of MoSe_2_-Ni_2_P hybrid nanostructures. (c) TEM and (d) HRTEM images of MoSe_2_-Co_2_P hybrid nanostructures.

The as-prepared MoSe_2_ nanosheet-assembled nanospheres were then used as templates for the growth of hybrid nanostructures. For example, Ni_2_P nanoparticles have been grown on the surface of MoSe_2_ nanospheres to form the MoSe_2_-Ni_2_P hybrid nanostructures (see Materials and Methods for details). From the TEM image (Figure 2(a)), it can be seen that the Ni_2_P nanoparticles are coated on the surface of MoSe_2_ nanospheres. Based on the XRD analysis ([Supplementary-material supplementary-material-1]), the as-obtained hybrid nanostructure is composed of crystalline 2H phase of MoSe_2_ (JCPDS No. 15-0029) and hexagonal phase of Ni_2_P (JCPDS No. 65-1989, *a* = 5.859 *Å*, *c* = 3.382 *Å*). The HRTEM image (Figure 2(b)) further confirms that the Ni_2_P nanoparticles are highly crystalline with a lattice spacing of 2.31 Å attributing to the (111) planes of the hexagonal phase Ni_2_P. The measured lattice distance of 6.89 Å corresponds to the (002) plane of 2H phase MoSe_2_. The size of Ni_2_P nanoparticles in the hybrid nanostructures is 8.7 ± 1.3 nm ([Supplementary-material supplementary-material-1]). The energy-dispersive X-ray spectroscopy (EDS) spectrum ([Supplementary-material supplementary-material-1]) shows that the estimated molar ratios of Mo/Se and Ni/P are 1/2.2 and 1.6/1, respectively, close to the calculated stoichiometric ratios. The corresponding EDS elemental mapping ([Supplementary-material supplementary-material-1]) confirms the homogeneous distribution of Mo, Se, Ni, and P, further revealing the successful growth of Ni_2_P on the surface of MoSe_2_ nanospheres.

Importantly, our method is general, which can be used to grow other nanostructures on MoSe_2_ nanospheres. For example, Co_2_P nanoparticles can also be grown on the surface of MoSe_2_ nanospheres to form MoSe_2_-Co_2_P hybrid nanostructures (Figure 2(c)). The measured lattice fringes of 6.89 Å and 2.23 Å match well with the (002) planes of MoSe_2_ and (121) planes of Co_2_P, respectively. The size of Co_2_P nanoparticles in the hybrid nanostructures is 7.5 ± 1.4 nm ([Supplementary-material supplementary-material-1]). The XRD analysis ([Supplementary-material supplementary-material-1]) demonstrates the coexistence of MoSe_2_ (JCPDS No. 15-0029) and Co_2_P (JCPDS No. 32-0306, orthorhombic, *a* = 5.6465 *Å*, *b* = 6.6099 *Å*, *c* = 3.513 *Å*). The molar ratios of Mo/Se and Co/P are 1/1.5 and 2.2/1, respectively, as characterized by EDS ([Supplementary-material supplementary-material-1]). The presence of Mo, Se, Co, and P and their homogeneous distributions can be clearly observed in the EDS elemental mapping of MoSe_2_-Co_2_P hybrid nanostructures ([Supplementary-material supplementary-material-1]).

**Figure 3:**
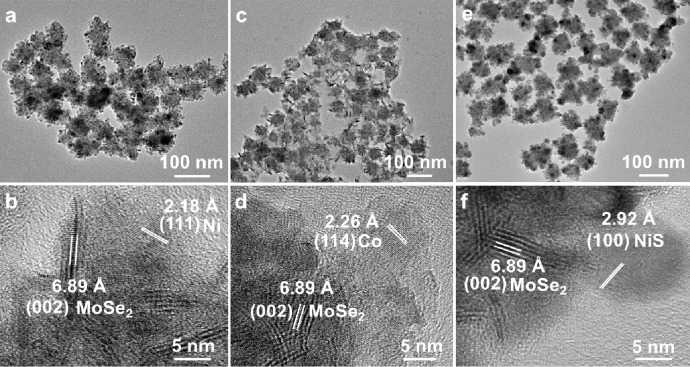
TEM and HRTEM measurements of the MoSe_2_-Ni, MoSe_2_-Co, and MoSe_2_-NiS hybrid nanostructures. (a) TEM and (b) HRTEM images of the MoSe_2_-Ni hybrid nanostructures. (c) TEM and (d) HRTEM images of the MoSe_2_-Co hybrid nanostructures. (e) TEM and (f) HRTEM images of the MoSe_2_-NiS hybrid nanostructures.

Besides Ni_2_P and Co_2_P nanoparticles, metallic Ni and Co nanoparticles have also been successfully grown on the surface of MoSe_2_ nanospheres (see Materials and Methods for details).  Figure 3(a) shows that the Ni nanoparticles have been grown on the surface of MoSe_2_ nanospheres to form the MoSe_2_-Ni hybrid nanostructures. The size of Ni nanoparticles in the hybrid nanostructures is 7.4 ± 1.5 nm ([Supplementary-material supplementary-material-1]). In the HRTEM image (Figure 3(b)), the measured interplanar distances between lattice fringes are estimated to be 6.89 Å and 2.18 Å, which match well with the (002) planes of MoSe_2_ and (111) planes of Ni, respectively. The cubic Ni (JCPDS No. 01-1258, *a* = 3.54 *Å*) was confirmed by the XRD pattern ([Supplementary-material supplementary-material-1]). [Supplementary-material supplementary-material-1] confirms the magnetic property of Ni nanoparticles in the prepared MoSe_2_-Ni hybrid nanostructures. The EDS ([Supplementary-material supplementary-material-1]) and corresponding elemental mapping ([Supplementary-material supplementary-material-1]) demonstrate the presence of Mo, Se, and Ni and their homogeneous distribution. Using the similar method, the MoSe_2_-Co hybrid nanostructure can also be prepared (Figure 3(c)). The corresponding HRTEM image reveals that the observed lattice spacing for MoSe_2_ (002) planes and Co (114) planes are 6.89 Å and 2.26 Å, respectively. The size of Co nanoparticles in the hybrid nanostructures is 6.4 ± 1.4 nm ([Supplementary-material supplementary-material-1]). The XRD pattern identifies that the MoSe_2_ (JCPDS No. 15-0029) and hexagonal phase Co (JCPDS No. 65-9722, *a* = 8.288 *Å*, *c* = 10.542 *Å*) coexist ([Supplementary-material supplementary-material-1]). [Supplementary-material supplementary-material-1] confirms the magnetic property of Co nanoparticles in the prepared MoSe_2_-Co hybrid nanostructures. Furthermore, the presence of Mo, Se, and Co and their homogeneous distributions have been shown in the EDS spectrum ([Supplementary-material supplementary-material-1]) and corresponding elemental mapping ([Supplementary-material supplementary-material-1]).

Moreover, after the sulfurization of the MoSe_2_-Ni hybrid nanostructure, the MoSe_2_-NiS hybrid nanostructure can also be synthesized by adding sulfur precursor to the reaction solution (see Materials and Methods for details). The as-synthesized nanostructure was characterized by XRD ([Supplementary-material supplementary-material-1]). The XRD analysis of the final product reveals a mixture of hexagonal MoSe_2_ (JCPDS No. 15-0029) and hexagonal NiS (JCPDS No. 02-1273, *a* = 3.440 *Å*, *c* = 5.350 *Å*). The TEM image (Figure 3(e)) shows that the NiS nanoparticles are uniformly coated on the surface of the MoSe_2_ nanospheres. The size of NiS nanoparticles in the hybrid nanostructures is 11.7 ± 1.4 nm ([Supplementary-material supplementary-material-1]). As shown in the corresponding HRTEM image, the lattice spacing of 6.89 Å corresponds to the (002) planes of MoSe_2_, while the other one of 2.92 Å is attributed to the (100) planes of NiS (Figure 3(f)). The EDS spectrum in [Supplementary-material supplementary-material-1] shows that the Mo/Se and Ni/S molar ratios are estimated to 1/2.4 and 1/1, respectively, close to the corresponding stoichiometric ratios. The EDS elemental mapping ([Supplementary-material supplementary-material-1]) further demonstrates the homogeneous distributions of Mo, Se, Ni, and S in the as-prepared nanostructure.

**Figure 4:**
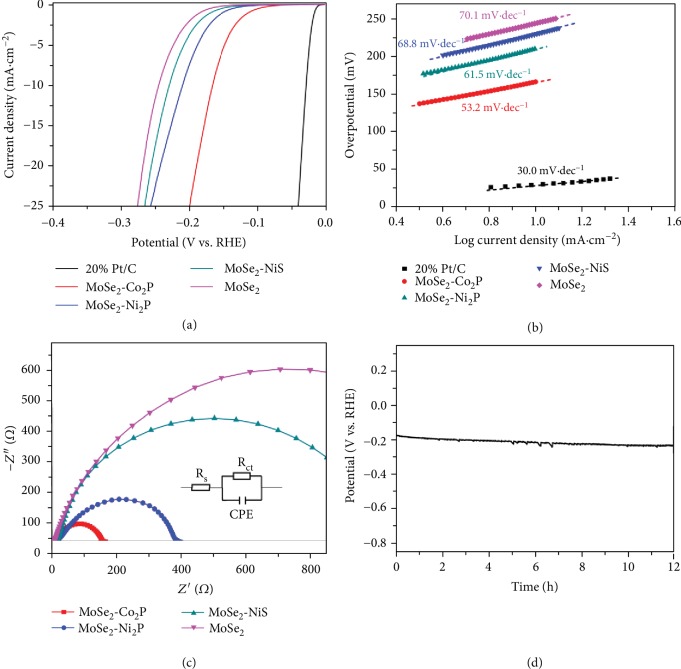
HER electrochemical performances of the hybrid nanostructures. (a) Polarization curves of the 20 wt% Pt/C, MoSe_2_-Co_2_P, MoSe_2_-Ni_2_P, and MoSe_2_-NiS hybrid nanostructures, and MoSe_2_ nanosphere with iR correction. (b) The corresponding Tafel plots. (c) Nyquist plots of MoSe_2_-Co_2_P, MoSe_2_-Ni_2_P, and MoSe_2_-NiS hybrid nanostructures and MoSe_2_ nanosphere collected at a bias voltage of -150 mV. Inset: the equivalent circuit used for fitting the Nyquist plots. (d) Chronopotentiometry response for MoSe_2_-Co_2_P hybrid nanostructures at 10 mA cm^−2^ for 12 h.

As a proof-of-concept application, the aforementioned synthesized hybrid nanostructures, including MoSe_2_-Ni_2_P, MoSe_2_-Co_2_P, and MoSe_2_-NiS, were used as catalysts for the electrochemical HER. The electrochemical HER activities were tested in 0.5 M H_2_SO_4_ aqueous solution using a standard three-electrode system.  Figure 4(a) shows typical polarization curves of these as-prepared hybrid nanostructures and the 20 wt% Pt/C. The MoSe_2_ nanosphere shows the lowest HER activity with overpotential of 245 mV at 10 mA cm^−2^. After growth of NiS, Ni_2_P, and Co_2_P on the MoSe_2_ nanospheres, the catalytic activities of hybrid nanostructures were significantly enhanced. The overpotentials at 10 mA cm^−2^ are 231, 211, and 167 mV for the MoSe_2_-NiS, MoSe_2_-Ni_2_P, and MoSe_2_-Co_2_P hybrid nanostructures, respectively. Furthermore, the Tafel slopes were used to evaluate the HER kinetics. As shown in Figure 4(b), the measured Tafel slope of Pt/C is 30.0 mV dec^−1^, close to the reported value [10–12]. The Tafel slope of MoSe_2_ nanospheres is 70.1 mV/dec, which decreases to 68.8, 61.5, and 53.2 mV/dec for MoSe_2_-NiS, MoSe_2_-Ni_2_P, and MoSe_2_-Co_2_P hybrid nanostructures, respectively. In addition, as shown in Figure 4(c), the electrochemical impedance spectroscopy (EIS) reveals that these hybrid nanostructures exhibit a faster electron/charge transfer rate than does the MoSe_2_ nanosphere ([Supplementary-material supplementary-material-1]), suggesting that the growth of NiS, Ni_2_P, and Co_2_P on MoSe_2_ nanospheres can significantly enhance the electrical conductivity. The good electron-transfer kinetics is important for electrocatalysts to exhibit high activity [55–57]. Note that the performance of MoSe_2_-Co_2_P is comparable to or even better than those previously reported similar materials for the acid HER ([Supplementary-material supplementary-material-1]).

It is important to understand the mechanism for the improved HER performance of MoSe_2_-Co_2_P hybrid nanostructures. First, the more postgrowth nanostructures mean the more exposure of active sites, enabling a high utilization ratio of catalysts [58–61]. The effect of the surface area on HER was evaluated through the electrochemical double-layer capacitance (*C*_dl_, [Supplementary-material supplementary-material-1]). The fitted *C*_dl_ of MoSe_2_-Co_2_P is close to that of MoSe_2_, revealing that the promoted HER performance should be derived from the enhanced intrinsic activity rather than the increased electrochemical active specific area or active sites. On the other hand, the *R*_ct_ value of the MoSe_2_-Co_2_P hybrid nanostructures is smaller than the pure MoSe_2_ nanospheres ([Supplementary-material supplementary-material-1]), indicating the fastest charge transfer process. These results clearly show the crucial role of the synergetic effect between MoSe_2_ and Co_2_P in MoSe_2_-Co_2_P hybrid nanostructures, which is responsible for the enhanced electrochemical hydrogen evolution.

The stability of the MoSe_2_-Co_2_P hybrid nanostructures for HER catalysis was tested using the chronopotentiometry method. As shown in Figure 4(d), only a slight voltage drop is observed even after 12 h electrolysis of water at current density of 10 mA cm^−2^, indicating the excellent stability of the MoSe_2_-Co_2_P hybrid nanostructures. Moreover, after the stability test, the polarization curve only shows a slight negative shift ([Supplementary-material supplementary-material-1]) as compared with the initial one. These results imply the long-term stability of MoSe_2_-Co_2_P catalyst for HER.

## 3. Discussion

In summary, by using the synthesized MoSe_2_ nanospheres as templates, nickel and cobalt-based nanomaterials can be synthesized on them to form a series of the MoSe_2_ nanosphere-based hybrid nanostructures, including MoSe_2_-Ni_2_P, MoSe_2_-Co_2_P, MoSe_2_-Ni, MoSe_2_-Co, and MoSe_2_-NiS. Importantly, as a proof-of-concept application, when used as electrocatalysts, the MoSe_2_-Ni_2_P, MoSe_2_-Co_2_P, and MoSe_2_-NiS hybrid nanostructures exhibited high HER activities in acidic environment. Among them, the MoSe_2_-Co_2_P hybrid nanostructures exhibit excellent stability and highest HER activity with an overpotential of 167 mV at 10 mA cm^−2^. We believe that as a general method for hybridizing layered TMD nanostructures with transition metal chalcogenide/phosphide nanocrystals, this strategy is applicable for growth of many other nanomaterials to form hybrid nanostructures with enhanced electrochemical activity and stability.

## 4. Materials and Methods

### 4.1. Chemicals

Molybdenum chloride (MoCl_5_) was purchased from Alfa Aesar. Sulfur, selenium, nickel acetylacetonate (Ni(acac)_2_), cobalt acetylacetonate (Co(acac)_2_), trioctylphosphine (TOP), octadecene (ODE), stearic acid (SA), oleylamine (OLA), and toluene were purchased from Aldrich. All the chemicals were used as received without further purification.

### 4.2. Synthesis of MoSe_2_ Nanosheet-Assembled Nanospheres

In a typical procedure, 0.5 mmol of MoCl_5_, 1 g of SA, and 9 mL of ODE were added into a 100 mL three-necked flask. The aforementioned mixture, denoted as solution A, was degassed under a vacuum at 120°C for 10 min. Then, the temperature was heated up to 300°C under nitrogen. At the same time, 1 mmol of selenium powder was dissolved into 2 mL of ODE at 250°C, which was denoted as solution B. Solution B was cooled to 130°C and subsequently injected into solution A when the temperature of mixed solution finally reached 300°C, which was then kept at 300°C for 30 min. After it was cooled down to room temperature, the product, i.e., MoSe_2_ nanospheres, was collected by centrifugation (10000 rpm, 5 min) and washed several times with toluene and acetone (technical grade) before further characterization. All products in this work were collected and washed by the same method.

### 4.3. Synthesis of MoSe_2_-Ni_2_P, MoSe_2_-Co_2_P, MoSe_2_-Ni, MoSe_2_-Co, and MoSe_2_-NiS Hybrid Nanostructures

In order to synthesize the MoSe_2_-Ni_2_P hybrid nanostructures, the freshly prepared MoSe_2_ nanospheres were redispersed into 10 mL of OLA containing 0.5 mmol Ni(acac)_2_ and 2 mL TOP. The mixture was degassed under a vacuum at 110°C for 10 min. Then, the temperature was increased to 320°C under nitrogen atmosphere and kept for 1 h before cooling down to room temperature. The MoSe_2_-Co_2_P hybrid nanostructures were synthesized by the same method, except that Co(acac)_2_ was used instead of Ni(acac)_2_. The preparation procedure of MoSe_2_-Ni and MoSe_2_-Co hybrid nanostructures was the same as the aforementioned procedure except that the reaction temperature was changed to 220°C from 320°C. For the synthesis of MoSe_2_-NiS hybrid nanostructures, the procedure was the same as that used for synthesis of MoSe_2_-Ni hybrid nanostructures, except that after the reaction was proceeded at 220°C for 1 h, 2 mL of sulfur powder (0.5 mmol) in oleylamine (OLA) was injected. The resulting mixed solution was kept at 220°C for another 1 h.

### 4.4. Characterization

The XRD were recorded on a Bruker D8 diffractometer (German) with a slit of 1/2° at a scanning rate of 2° min^−1^, using Cu K*_α_* radiation (*λ* = 1.5406 *Å*). TEM, HRTEM, and EDS mapping characterizations were performed on JEOL 2010F (Japan) and JEOL 2100F (Japan) with an acceleration voltage of 200 kV.

### 4.5. Electrode Preparation

Experimentally, 4 mg of acetylene black was first mixed with 5 mL of hexane and then sonicated to form a uniform suspension. Then, 8 mg of MoSe_2_, MoSe_2_-Ni_2_P, MoSe_2_-Co_2_P, or MoSe_2_-NiS in hexane was added into acetylene black suspension under sonication for 1 h. The mixed catalysts were separated by centrifugation, then washed with ethanol. The prepared catalysts were annealed at 400°C for 30 min under a flow 5% H_2_/Ar to remove surfactants. 4 mg of the respective catalyst powders was dispersed in 1 mL of 1 : 1 (*v*/*v*) water/ethanol mixed solvents under ultrasonication for 1 h. 5 *μ*L of the resulting solution was dropped onto the surface of a cleaned glassy carbon electrode by a microliter syringe and dried at room temperature. After drying at room temperature, the surface of the catalyst-based electrode was covered by 5 *μ*L of 1% Nafion solution.

### 4.6. Electrochemical Measurements

All the electrochemical experiments were performed on an electrochemical workstation (CHI 760C, CH Instruments Inc., USA), using a conventional three-electrode system, i.e., a Hg/Hg_2_Cl_2_ (saturated KCl, SCE) reference electrode, a carbon rod counter electrode, and the prepared working electrode. 0.5 M H_2_SO_4_ aqueous solution was used as the electrolyte throughout the experiments. Before the electrochemical measurement, the electrolyte was degassed by bubbling N_2_ for at least 30 min. The polarization curves were obtained by sweeping the potential from 0 to -0.6 V (vs. SCE) at a sweep rate of 2 mV s^−1^. Electrochemical impedance spectroscopy (EIS) was recorded in 0.5 M H_2_SO_4_ aqueous solution using an alternating current (AC) voltage of 5 mV and direct current (DC) voltage of -0.15 V (vs. RHE) within the frequency range from 100 kHz to 0.1 Hz. Voltage-time responses were monitored by chronopotentiometry measurement at 10 mA cm^−2^ for 12 h.

## Supplementary Materials

Supplementary Materials Figure S1: XRD pattern of the as-prepared MoSe_2_ nanosheet-assembled nanospheres. Figure S2: XRD pattern of the as-prepared MoSe_2_-Ni_2_P nanostructures. Figure S3: statistical analysis of the size of 50 Ni_2_P nanoparticles measured from HRTEM images. Figure S4: EDS spectrum of the as-prepared MoSe_2_-Ni_2_P nanostructures. Figure S5: EDS mapping of the as-prepared MoSe_2_-Ni_2_P hybrid nanostructures. Figure S6: statistical analysis of the size of 50 Co_2_P nanoparticles measured from HRTEM images. Figure S7: XRD pattern of the as-prepared MoSe_2_-Co_2_P hybrid nanostructures. Figure S8: EDS spectrum of the as-prepared MoSe_2_-Co_2_P hybrid nanostructures. Figure S9: EDS elemental mapping of the as-prepared MoSe_2_-Co_2_P hybrid nanostructures. Figure S10: statistical analysis of the size of 50 Ni nanoparticles measured from HRTEM images. Figure S11: (a) XRD pattern of the as-prepared MoSe_2_-Ni hybrid nanostructures. (b) Top: photograph of MoSe_2_-Ni in toluene. Bottom: photograph showing the magnetic property of MoSe_2_-Ni in toluene in the presence of a magnet. Figure S12: EDS spectrum of the as-prepared MoSe_2_-Ni hybrid nanostructures. Figure S13: EDS elemental mapping of the as-prepared MoSe_2_-Ni hybrid nanostructures. Figure S14: statistical analysis of the size of 50 Co nanoparticles measured from HRTEM images. Figure S15: (a) XRD pattern of the as-prepared MoSe_2_-Co hybrid nanostructures. (b) Top: photograph of MoSe_2_-Co in toluene. Bottom: photograph showing the magnetic property of MoSe_2_-Co in toluene in the presence of a magnet. Figure S16: EDS spectrum of the as-prepared MoSe_2_-Co hybrid nanostructures. Figure S17: EDS elemental mapping of the as-prepared MoSe_2_-Co hybrid nanostructures. Figure S18: XRD pattern of the as-prepared MoSe_2_-NiS hybrid nanostructures. Figure S19: statistical analysis of the size of 50 NiS nanoparticles measured from HRTEM images. Figure S20: EDS spectrum of the as-prepared MoSe_2_-NiS hybrid nanostructures. Figure S21: EDS elemental mapping of the as-prepared MoSe_2_-NiS hybrid nanostructures. Figure S22: cyclic voltammetry curves of (a) MoSe_2_-Co_2_P hybrid nanostructures and (b) MoSe_2_ nanosphere in the region of 0.15-0.25 V vs. RHE. (c) The differences in current density at 0.20 V vs. RHE plotted against scan rate fits to a linear regression. Figure S23: polarization curves of MoSe_2_-Co_2_P hybrid nanostructures before and after 12 h-HER tests at 10 mA cm^−2^. Table S1: the charge transfer resistances (*R*_ct_) and constant phase elements (CPEs) of the prepared electrocatalysts. Table S2: HER activities of the MoSe_2_-Co_2_P hybrid nanostructures and the reported electrocatalysts.
