# Supplementary material for: A General Method for the Synthesis of Hybrid Nanostructures Using MoSe2 Nanosheet-Assembled Nanospheres as Templates
Source: Research (Wash D C). 2019 Nov 14;2019:6439734. doi: 10.34133/2019/6439734 (PMC6944485; doi:10.34133/2019/6439734)
Supplement: Supplementary Materials — Figure S1: XRD pattern of the as-prepared MoSe2 nanosheet-assembled nanospheres. Figure S2: XRD pattern of the as-prepared MoSe2-Ni2P nanostructures. Figure S3: statistical analysis of the size of 50 Ni2P nanoparticles measured from HRTEM images. Figure S4: EDS spectrum of the as-prepared MoSe2-Ni2P nanostructures. Figure S5: EDS mapping of the as-prepared MoSe2-Ni2P hybrid nanostructures. Figure S6: statistical analysis of the size of 50 Co2P nanoparticles measured from HRTEM images. Figure S7: XRD pattern of the as-prepared MoSe2-Co2P hybrid nanostructures. Figure S8: EDS spectrum of the as-prepared MoSe2-Co2P hybrid nanostructures. Figure S9: EDS elemental mapping of the as-prepared MoSe2-Co2P hybrid nanostructures. Figure S10: statistical analysis of the size of 50 Ni nanoparticles measured from HRTEM images. Figure S11: (a) XRD pattern of the as-prepared MoSe2-Ni hybrid nanostructures. (b) Top: photograph of MoSe2-Ni in toluene. Bottom: photograph showing the magnetic property of MoSe2-Ni in toluene in the presence of a magnet. Figure S12: EDS spectrum of the as-prepared MoSe2-Ni hybrid nanostructures. Figure S13: EDS elemental mapping of the as-prepared MoSe2-Ni hybrid nanostructures. Figure S14: statistical analysis of the size of 50 Co nanoparticles measured from HRTEM images. Figure S15: (a) XRD pattern of the as-prepared MoSe2-Co hybrid nanostructures. (b) Top: photograph of MoSe2-Co in toluene. Bottom: photograph showing the magnetic property of MoSe2-Co in toluene in the presence of a magnet. Figure S16: EDS spectrum of the as-prepared MoSe2-Co hybrid nanostructures. Figure S17: EDS elemental mapping of the as-prepared MoSe2-Co hybrid nanostructures. Figure S18: XRD pattern of the as-prepared MoSe2-NiS hybrid nanostructures. Figure S19: statistical analysis of the size of 50 NiS nanoparticles measured from HRTEM images. Figure S20: EDS spectrum of the as-prepared MoSe2-NiS hybrid nanostructures. Figure S21: EDS elemental mapping of the as-prepared MoSe2- [file 6439734.f1.docx]

Supplementary Materials for *Research*

# A general method for the synthesis of hybrid nanostructures using MoSe_2_ nanosheet-assembled nanospheres as templates

Shikui Han^†,1,2^, Kai Zhou^†,2,3,4^, Yifu Yu^2^, Chaoliang Tan^2^, Junze Chen^2^, Ying Huang^2^, Qinglang Ma^2^, Ye Chen^2^, Hongfei Cheng^2^, Weijia Zhou^4^, and Hua Zhang^2,5*^

^1^*Key Laboratory of Advanced Catalytic Materials and Reaction Engineering, School of Chemistry and Chemical Engineering, Hefei University of Technology, Hefei 230009, China.*

^2^*Center for Programmable Materials, School of Materials Science and Engineering, Nanyang Technological University, 50 Nanyang Avenue, Singapore 639798, Singapore.*

^3^*Center for Advanced Analytical Science, School of Chemistry and Chemical Engineering, Guangzhou University, Guangzhou 510006, China.*

^4^*New Energy Research Institute, School of Environment and Energy, South China University of Technology, Guangzhou Higher Education Mega Center, Guangzhou 510006, China.*

^5^*Department of Chemistry, City University of Hong Kong, Tat Chee Avenue, Kowloon, Hong Kong, China.*

^†^These authors contributed equally to this work.

*Correspondence should be addressed to the author. E-mail: [hzhang@ntu.edu.sg](mailto:hzhang@ntu.edu.sg), [hua.zhang@cityu.edu.cn](mailto:hua.zhang@cityu.edu.cn).

**Figure S1**. XRD pattern of the as-prepared MoSe_2_ nanosheet-assembled nanospheres.

**Figure S2**. XRD pattern of the as-prepared MoSe_2_-Ni_2_P nanostructures.

**Figure S3**. Statistical analysis of the size of 50 Ni_2_P nanoparticles measured from HRTEM images.

**Figure S4**. EDS spectrum of the as-prepared MoSe_2_-Ni_2_P nanostructures.

**Figure S5**. EDS mapping of the as-prepared MoSe_2_-Ni_2_P hybrid nanostructures.

**Figure S6**. Statistical analysis of the size of 50 Co_2_P nanoparticles measured from HRTEM images.

**Figure S7**. XRD pattern of the as-prepared MoSe_2_-Co_2_P hybrid nanostructures.

**Figure S8**. EDS spectrum of the as-prepared MoSe_2_-Co_2_P hybrid nanostructures.

**Figure S9**. EDS elemental mapping of the as-prepared MoSe_2_-Co_2_P hybrid nanostructures.

**Figure S10**. Statistical analysis of the size of 50 Ni nanoparticles measured from HRTEM images.

**Figure S11**. (a) XRD pattern of the as-prepared MoSe_2_-Ni hybrid nanostructures. (b) Top: Photograph of MoSe_2_-Ni in toluene. Bottom: Photograph showing the magnetic property of MoSe_2_-Ni in toluene in the presence of magnet.

**Figure S12**. EDS spectrum of the as-prepared MoSe_2_-Ni hybrid nanostructures.

**Figure S13**. EDS elemental mapping of the as-prepared MoSe_2_-Ni hybrid nanostructures.

**Figure S14**. Statistical analysis of the size of 50 Co nanoparticles measured from HRTEM images.

**Figure S15**. (a) XRD pattern of the as-prepared MoSe_2_-Co hybrid nanostructures. (b) Top: Photograph of MoSe_2_-Co in toluene. Bottom: Photograph showing the magnetic property of MoSe_2_-Co in toluene in the presence of magnet.

**Figure S16**. EDS spectrum of the as-prepared MoSe_2_-Co hybrid nanostructures.

**Figure S17**. EDS elemental mapping of the as-prepared MoSe_2_-Co hybrid nanostructures.

**Figure S18**. XRD pattern of the as-prepared MoSe_2_-NiS hybrid nanostructures.

**Figure S19**. Statistical analysis of the size of 50 NiS nanoparticles measured from HRTEM images.

**Figure S20**. EDS spectrum of the as-prepared MoSe_2_-NiS hybrid nanostructures.

**Figure S21**. EDS elemental mapping of the as-prepared MoSe_2_-NiS hybrid nanostructures.


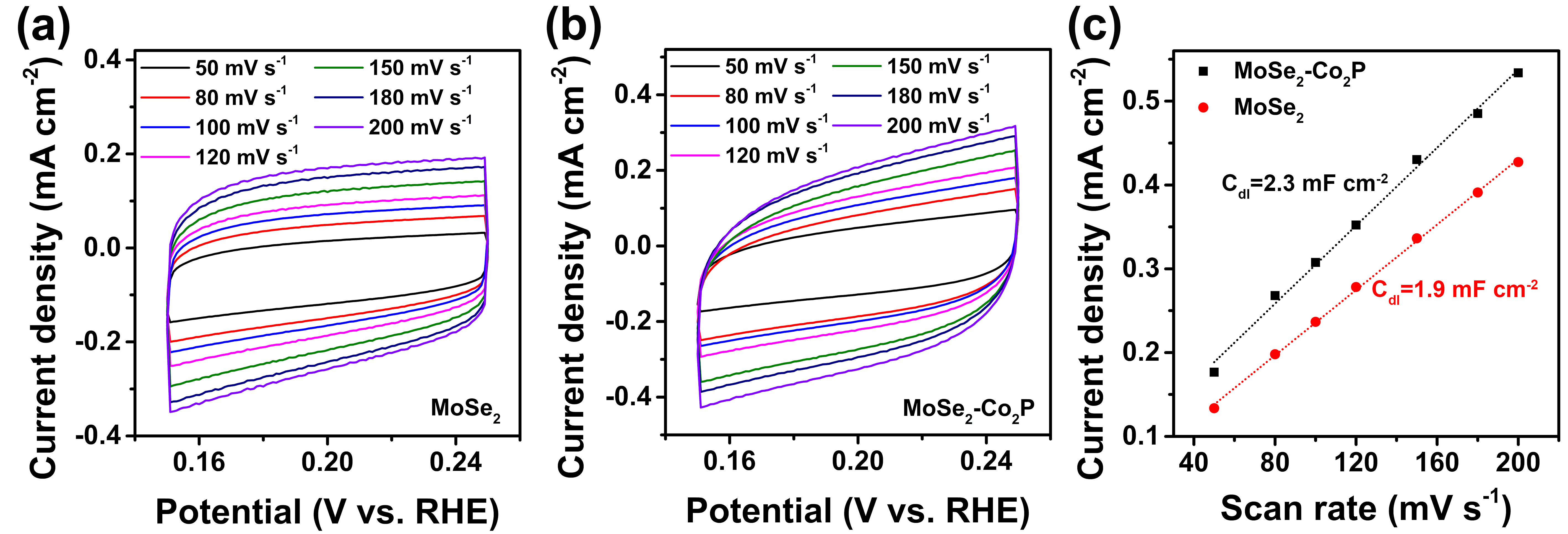


**Figure S22**. Cyclic voltammetry curves of (a) MoSe_2_-Co_2_P hybrid nanostructures, and (b) MoSe_2_ nanosphere in the region of 0.15-0.25 V vs. RHE. (c) The differences in current density at 0.20 V vs. RHE plotted against scan rate fits to a linear regression.


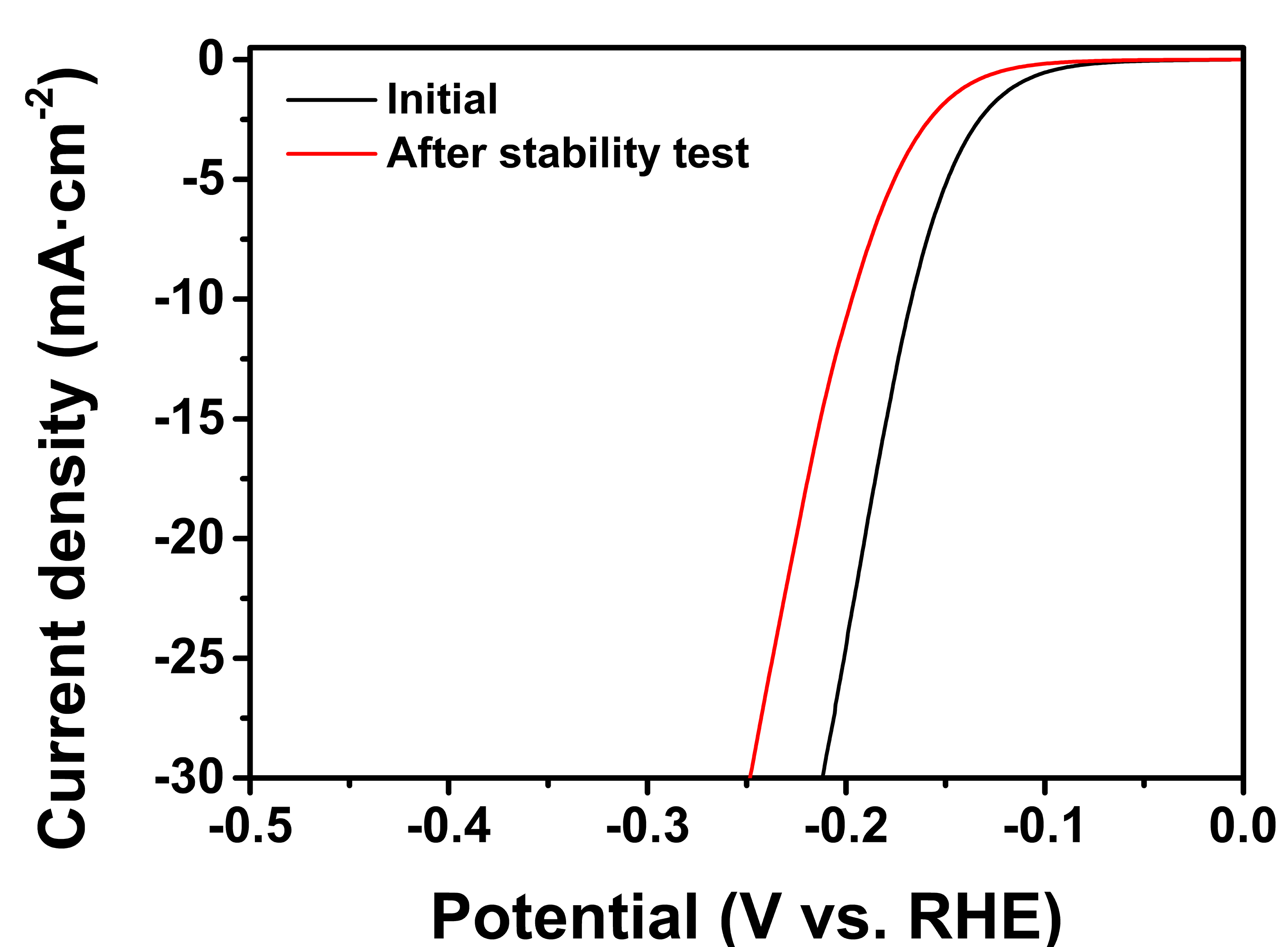


**Figure S23.** Polarization curves of MoSe_2_-Co_2_P hybrid nanostructures before and after 12 h-HER tests at 10 mA cm^-2^.

**Table S1**. The charge transfer resistances (Rct) and constant phase elements (CPEs) of the prepared electrocatalysts.

|  | MoSe_2_-Co_2_P | MoSe_2_-Ni_2_P | MoSe_2_-NiS | MoSe_2_ |
| --- | --- | --- | --- | --- |
| Rct (Ω) | 152.8 | 426.7 | 1184 | 1606 |
| CPE-P* | 0.77776 | 0.61255 | 0.76216 | 0.82679 |
| CPE-T* | 0.001191 | 0.00077424 | 0.0007352 | 0.0001708 |

*The constant phase elements (CPEs) can be expressed by the phase angle parameter (CPE-P) and the capacitance extent (CPE-T).

**Table S2**. HER activities of the MoSe_2_-Co_2_P hybrid nanostructures and the reported electrocatalysts

| Catalysts | Electrolyte | Overpotential (mV)  (10 mA cm^-1^) | Tafel slope (mV dec^-1^) | Ref. |
| --- | --- | --- | --- | --- |
| MoSe_2_-Co_2_P | 0.5 M H_2_SO_4_ | 167 | 53.2 | This work |
| MoSe_2_-NiSe | 0.5 M H_2_SO_4_ | 210 | 56 | [1] |
| MoSe_2_-rGO-CNT | 0.5 M H_2_SO_4_ | 240 | 53 | [2] |
| MoSe_2_ on vertical graphene arrays | 0.5 M H_2_SO_4_ | 190 | 62 | [3] |
| MoSe_2_/Graphene Hybrid Nanostructures | 0.5 M H_2_SO_4_ | 195 | 67 | [4] |
| SnO_2_@MoSe_2_ | 0.5 M H_2_SO_4_ | 174 | 51 | [5] |

**References**

[1] X. Zhou, Y. Liu, H. Ju, B. Pan, J. Zhu, T. Ding, C. Wang, Q. Yang, "Design and Epitaxial Growth of MoSe_2_–NiSe Vertical Heteronanostructures with Electronic Modulation for Enhanced Hydrogen Evolution Reaction," *Chemistry of Materials*, vol. 28, no. 6, pp. 1838-1846, 2016.

[2] G. D. Park, J. H. Kim, S.-K. Park, Y. C. Kang, "MoSe_2_ Embedded CNT-Reduced Graphene Oxide Composite Microsphere with Superior Sodium Ion Storage and Electrocatalytic Hydrogen Evolution Performances," *ACS Applied Materials & Interfaces*, vol. 9, no. 12, pp. 10673-10683, 2017.

[3] S. Deng, Z. Yu, Y. Zeng, Y. Wang, Z. Yao, F. Yang, S. Lin, *et al.*, "Directional Construction of Vertical Nitrogen‐Doped 1T‐2H MoSe_2_/Graphene Shell/Core Nanoflake Arrays for Efficient Hydrogen Evolution Reaction," *Advanced Materials*, vol. 29, no. 21, p. 1700748, 2017.

[4] Z. Liu, N. Li, H. Zhao, Y. Du, "Colloidally Synthesized MoSe_2_/Graphene Hybrid Nanostructures as Efficient Electrocatalysts for Hydrogen Evolution," *Journal of Materials Chemistry A*, vol. 3, no. 39, pp. 19706-19710, 2015.

[5] Y. Huang, Y.-E. Miao, J. Fu, S. Mo, C. Wei, T. Liu, "Perpendicularly Oriented Few-Layer MoSe_2_ on SnO_2_ Nanotubes for Efficient Hydrogen Evolution Reaction," *Journal of Materials Chemistry A*, vol. 3, no. 31, pp. 16263-16271, 2015.
